# Scaffold-based lung tumor culture on porous PLGA microparticle substrates

**DOI:** 10.1371/journal.pone.0217640

**Published:** 2019-05-31

**Authors:** Aneetta E. Kuriakose, Wenjing Hu, Kytai T. Nguyen, Jyothi U. Menon

**Affiliations:** 1 Bioengineering Department, University of Texas at Arlington, Arlington, Texas, United States of America; 2 Graduate Biomedical Engineering Program, UT Southwestern Medical Center, Dallas, Texas, United States of America; 3 Progenitec Inc., Arlington, Texas, United States of America; 4 Department of Biomedical and Pharmaceutical Sciences, College of Pharmacy, University of Rhode Island, Kingston, Rhode Island, United States of America; Technische Universitat Dresden, GERMANY

## Abstract

Scaffold-based cancer cell culture techniques have been gaining prominence especially in the last two decades. These techniques can potentially overcome some of the limitations of current three-dimensional cell culture methods, such as uneven cell distribution, inadequate nutrient diffusion, and uncontrollable size of cell aggregates. Porous scaffolds can provide a convenient support for cell attachment, proliferation and migration, and also allows diffusion of oxygen, nutrients and waste. In this paper, a comparative study was done on porous poly (lactic-co-glycolic acid) (PLGA) microparticles prepared using three porogens—gelatin, sodium bicarbonate (SBC) or novel poly N-isopropylacrylamide [PNIPAAm] particles, as substrates for lung cancer cell culture. These fibronectin-coated, stable particles (19–42 μm) supported A549 cell attachment at an optimal cell seeding density of 250,000 cells/ mg of particles. PLGA-SBC porous particles had comparatively larger, more interconnected pores, and favored greater cell proliferation up to 9 days than their counterparts. This indicates that pore diameters and interconnectivity have direct implications on scaffold-based cell culture compared to substrates with minimally interconnected pores (PLGA-gelatin) or pores of uniform sizes (PLGA-PMPs). Therefore, PLGA-SBC-based tumor models were chosen for preliminary drug screening studies. The greater drug resistance observed in the lung cancer cells grown on porous particles compared to conventional cell monolayers agrees with previous literature, and indicates that the PLGA-SBC porous microparticle substrates are promising for *in vitro* tumor or tissue development.

## Introduction

The practice of tissue and cell culture has been in existence as early as 1885 when Wilhelm Roux demonstrated that the medullary plate of a chick embryo can be maintained on glass plates with warm saline solution [1, 2]. Since then, cells have been traditionally cultured *in vitro* on two-dimensional (2D) polystyrene or glass surfaces. 2D cell culture models are still in use in pharmacology today for drug screening and cytocompatibility studies. However, these conventional 2D systems differ from *in vivo* tissues in cell surface receptor expression, extracellular matrix synthesis, cell density, and metabolic functions [3]. They are also unable to develop hypoxia or mimic the cell arrangement seen in different parts of the tissues and tumors [4]. Further, studies have shown that tumor cell monolayers grown on tissue culture plates develop a non-natural morphology, which could be a major factor affecting their responses to drugs [5]. According to recent reports, the promising effects of therapeutic agents in *in vitro* 2D cell culture systems have not translated into successful results in animals, and in humans. Only about 5% of the chemotherapeutic agents that showed promising preclinical activity have demonstrated significant therapeutic efficacy in phase III clinical trials [6]. Therefore, there is a vital need for an *in vitro* cell culture model that mimics *in vivo* tissues more closely, for cancer drug screening and personalized medicine applications.

Several platforms for 3D cell culture have being investigated today and have demonstrated potential to recreate cancer microenvironment and drug responses similar to *in vivo* conditions. Scaffold-free methods such as spheroids formed by self-assembly of cells is one of the most common and versatile methods of culturing cells in 3D [7]. Spheroids can recapitulate the 3D architecture of tissues and mimic the physiological barriers that affects drug delivery *in vivo*. However, the main challenge in using spheroids as efficacy testing models is their inability to form and maintain spheroids of uniform diameters [8]. Although this limitation can be overcome to some extent using microwells, specialized methods and facilities may be required for microwell generation. Furthermore, microwells are not suitable for long-term culture as cells can outgrow the space available in the wells [9]. Conventional methods including hanging drop are able to define the sizes of spheroids, but these procedures are extremely labor intensive and time consuming [10]. Magnetic levitation has been studied to form complex *in vitro* cell structures, however premature release of the magnetic micro/nanoparticles had raised toxicity concerns due to which approaches for improved magnet-based cell assembly are being investigated [11]. Another approach employs hydrogels embedded with tumor cells, but the spatial distribution of cells within the gels are not uniform resulting in variations between batches. Similar challenge is posed by large polymeric scaffolds where cells outside would be exposed to nutrients and oxygen, while cells within the scaffold may become necrotic quickly due to limited availability of resources essential for their growth [12, 13]. Bioprinting has been gaining prominence as it can provide spatial control for *in vitro* model development [14], however this method requires specialized equipment such as bioprinters and bioreactors which may raise the cost and reduce feasibility for high throughput screening [9]. In consideration of these challenges, biodegradable microparticles (MPs) offers a better alternative both to 2D and existing scaffold-free methods, as they provide large surface area suitable for cell attachment and long-term culture for tumor ECM deposition. They can also be used to generate organized cell arrangements *in vitro* according to the disease or tissue being studied, which is an advantage over 2D and several scaffold-free cell models [15]. Several natural (alginate [16], collagen [17], hyaluronic acid [18], basement membrane matrix [19]) and synthetic (poly(lactic acid-co-glycolic acid) [3], polycaprolactone [18], polyethylene glycol [20], polylactic acid [21]) polymer-based particles have been utilized to develop *in vitro* cancer models for various cancer studies.

In tissue engineered scaffolds and microparticles, porosity is an important parameter to be considered, in order to ensure high levels of cell density and viability by facilitating effective transfer of nutrients/oxygen and metabolic wastes during the culture [22]. Porous scaffolds tend to resemble the arrangement of the extracellular matrix, which facilitates cell attachment and proliferation [23]. Such porous microspheres have also shown to have great potential as injectable cell carriers for tissue engineering and regenerative purposes [24, 25] as well as a scaffold for tumor modelling [23, 26]. Depending on the porogen incorporated into particles the porosity can be enhanced or tuned for the required application. Although porous polymeric microparticles have been characterized before for various tissue engineering applications, there have been no comparative studies to the authors’ knowledge on the effects of different porogens on pore diameter and distribution within microparticles, and how this influences the development of tissue engineered tumors and tissues. Therefore, the goal of this work is to develop and screen porous microparticles prepared using different porogens and choose the optimum substrate for scaffold-based lung tumor culture *in vitro*.

When compared with other types of cancers, lung cancer is by far the leading cause of death, regardless of gender or ethnicity [27]. Although diagnosis at early stage of lung cancer offers a favorable prognosis, most patients (~75%) have advanced disease at the time of diagnosis [28]. Significant research into the oncological management of late stage lung cancer have occurred in recent years, which have been supported by promising *in vitro* data. However these have not translated into successful clinical trials and lung cancer survival rate remains poor [28]. The chief issue here is the lack of preclinical models capable of predicting the behavior and effect of targeted therapies in humans [29]. So our aim is to facilitate such translation by developing methods for bench-top lung tumor culture using porous microparticles, for anti-cancer drug testing and to study cancer progression.

To develop the *in vitro* model, PLGA—an FDA-approved biocompatible and biodegradable polymer—was used to form the particles. We compared porous PLGA microparticles developed using three porogens namely gelatin, sodium bicarbonate (SBC) or PNIPAAm (poly (N-isopropylacrylamide)) based particles (PMPs). Gelatin has been widely employed as sacrificial material to template vascular networks or cellular compartments in scaffolds [30, 31]. In this case, pore diameter is determined by the size of gelatin/water emulsion formed during synthesis of PLGA particles. These water droplets when dispersed in the oil phase can gradually coalesce over time, leading to an increase in size for the droplets [32]. Therefore, controlled pore formation is difficult with this method. SBC on other hand is a gas porogen, which creates pores in the substrate by generating gas bubbles (carbon dioxide) in mildly acidic environment [33]. These gas bubbles trapped within the particles can be in contact with each other, leading to the formation of a highly interconnected pore structure in the particles [34]. Lastly, we introduce an innovative method to control the pore diameter using PMPs (diameter ~1μm) developed previously by our lab, as porogens [35]. Due to the temperature responsive properties of PNIPA at its lower critical solution temperature (LCST) [35, 36], PMPs will shrink and diffuse out of the PLGA, leaving behind a uniformly porous matrix. The use of temperature-sensitive particles as porogens to develop porous polymeric substrates for cell attachment has not been reported before.

## Experimental section

All chemicals, unless mentioned specifically, were purchased from Sigma-Aldrich (St. Louis, MO) and used without further purification. No human participants or vertebrate animals were used in this research. Immortalized A549 lung adenocarcinoma cell line was purchased from ATCC (CCL-185) for *in vitro* cell culture experiments. The cell culture protocols were approved by the Institutional Review Board (IRB) at the University of Texas at Arlington.

### Preparation of PLGA MPs

All polymeric MPs were prepared by a standard double emulsion method. The concentrations of the components used in each MP preparation were optimized so that the final particles will have relatively similar diameters for better comparison. The preparation technique for gelatin porogen-based particles was modified from a previously published work by Huang et al [37]. In brief, 37.5 mg of gelatin (extracted from bovine skin, type B) dispersed in 60 ml of 1% (w/v) Polyvinyl alcohol (PVA) was added dropwise to 5 ml of 2% (w/v) PLGA (Lakeshore Biomaterials, Birmingham, AL) in dichloromethane and vortexed. The resulting emulsion was added to 1% (w/v) PVA solution placed in ice water bath. Following overnight stirring to evaporate the organic solvent, gelatin leaching was done by keeping the particle suspension in a warm water bath at 45°C under gentle stirring for 4 hours. The porous particles were then isolated by washing three times with water, followed by centrifugation at 4000 rpm for 5 mins and lyophilization. Non-porous (control) PLGA microspheres were also prepared by the same method, excluding the gelatin addition and leaching.

The SBC porogen-based particles were prepared by modifying the protocol of Ke et al. [38]. Briefly, 500 μl of 1% (w/v) SBC solution was added dropwise to 2 ml of 5% (w/v) PLGA solution in DCM. This emulsion was then added dropwise to 60 ml of 0.1% (w/v) PVA solution and gently stirred overnight. SBC leaching was done by centrifuging the obtained particles in PBS solution at a pH of 3–4 at a speed of 1500 rpm for 30 min. Subsequently, the particles were washed twice in DI water, isolated by centrifugation (1500 rpm, 30 min) and lyophilized to obtain porous PLGA microspheres.

To prepare the PMPs porogens, first the PMPs were prepared by free radical polymerization based on protocol previously developed by our group [35, 36]. Briefly, 1.54 g N-isopropylacrylamide (NIPA), 26.2 mg methylene-bis-acrylamide (BIS) and 43.8 mg sodium dodecyl sulfate (SDS) dispersed in 90 ml DI water was purged with nitrogen gas following which ammonium persulfate (APS) and 500 μl of N,N,N,N-tetramethylethylenediamine (TEMED) were added. The reaction was allowed to occur in a nitrogen atmosphere for 5 hours, and the particles were purified by dialysis and collected by lyophilization. To prepare PMP porogen-based PLGA microspheres, 0.5 ml of 0.3% w/v PMP suspension in DI water was added dropwise to 5% (w/v) PLGA solution in chloroform. The solution was vortexed and added dropwise to a 0.1% (w/v) PVA solution. Following overnight stirring, the particles were centrifuged and lyophilized. PMPs were leached out of MPs by shaking the suspension at 37°C for 2 hours. Porous particles were separated by centrifugation at 2000 rpm for 5 min and lyophilized for further studies.

### Physical characterization

Particle morphology was observed using Scanning electron microscopy (SEM, Hitachi S-3000N, Hitachi, Pleasanton, CA). Briefly, 10 μl of the MP suspension air-dried on a coverslip was silver sputter-coated and inserted into the SEM instrument. Additionally, the average MPs size and pore diameter on the particles were measured from these SEM images using ImageJ software. We considered five different images of particles to determine the particle and pore diameter. A total of ten pores per particle surface was measured, to determine the pore diameter. Two independent observers measured the pore diameters using the same technique, to minimize observer bias. The surface charge on the particles was measured by dynamic light scattering (DLS) as described previously [39–41].

### Particle stability and degradation kinetics

To study stability of particles, the particles were dispersed in the 10% serum and incubated for 3 days at 37°C, and their size was evaluated at predetermined time points using DLS as described earlier [39, 42]. Particle degradation kinetics were studied by dispersing pre-weighed MPs in PBS and incubating while shaking at 37° C for 4 weeks. At predetermined time intervals, the particles were collected by centrifugation (4000 rpm, 5 min), and then lyophilized. The degradation profile was determined by calculating the residual weight of the particles over time, compared to their initial weight at day 0.

### *In vitro* cell studies

For *in vitro* studies, the porous particles were sterilized by overnight UV light exposure followed by immersion in 70% ethanol and washing with sterile phosphate buffered saline (PBS) and cell culture media. Serum proteins in cell culture media were expected to be adsorbed onto the porous MPs, which would provide a suitable surface for cell adhesion and proliferation [43]. Further, the well-plates were coated with 100 μl of 0.5% w/v agarose for all studies associated with MPs to ensure that cells do not adhere to the bottom of the wells.

#### Cell attachment and viability study

To determine optimum cell seeding density on MPs, A549 lung adenocarcinoma cells (ATCC, Manassas, VA) were seeded at varying densities of 15, 25, 35, 45, and 55 x 10^4^ cells per mg of particles per well of a 48 well plates. 0.2 mg of particles were added per well. The well-plates were incubated at 37°C in alternating shaking (30 rpm, 25 mins) and static conditions (25 mins) for the first 2 hours of incubation following which they were kept for shaking. After 24 hours, the samples were washed well with PBS to remove unattached cells, and particles were transferred to a new 48-well plate. MTS assays (Cell Titer96; PROMEGA, Madison, WI) were performed per manufacturer’s instructions and absorbance was read at 490 nm using a UV-Vis spectrophotometer. A standard curve was established with known cell numbers as described previously [44, 45], and absorbance readings were taken at the same time as the readings for the samples. Absorbance values of the samples were normalized against the cell standard to determine the cell number. To study the effect of surface coating on cellular attachment and proliferation, fibronectin-coated particles were prepared by sterilizing 5 mg of porous particles and placing them in 48 well plates with 5 ml of serum-free media containing 10 μg/ml fibronectin. These particles were then shaken at 37°C for 2 hours at 30 rpm following which cell attachment studies were conducted as described above. Cell attachment and viability was also visualized using Live/Dead staining (Invitrogen, Grand Island, NY).

For the SEM procedure, the particle-cell complexes were fixed using 2.5% glutaraldehyde followed by incubation at room temperature for 30 mins. After washing with PBS, the complexes were incubated with 1% osmium tetroxide for 1 hour. The samples were again washed and then gradually dehydrated in 50%, 75%, 95%, and 100% ethanol for 15 min. Hexamethyldisilazane (HMDS) was added to the samples that were then air-dried and visualized using SEM following sputter coating.

#### Cell proliferation study

Cells were seeded at the optimized density of 250,000 cells per mg of fibronectin-coated particles and were incubated by shaking at 37°C. At pre-determined time points (1, 3, 5, 7, and 9 days), MTS assays were carried out and the absorbance values were normalized against a cell standard. In parallel experiments, the cells were further visualized using either Live/Dead staining as mentioned above or SEM. Based on the results of *in vitro* studies, PLGA-SBC particles were chosen for further investigation.

#### *In vitro* drug screening study

The therapeutic efficacies of cancer drugs were studied *in vitro* using both 2D and our lung tumor cultures. In these studies, the optimized cell seeding density of 250,000 A549 cells per mg of fibronectin-coated particles was added to agarose-coated 48 well plates. The samples were incubated for 3 days at 37°C to ensure cell attachment and proliferation on the porous particles. For this study, we selected three FDA-approved lung cancer drugs Gemcitabine, Cisplatin, and Paclitaxel [46–49] and compared their effectiveness with three other drugs that showed higher therapeutic efficacy in preclinical trials (2D, xenograft models) for lung cancer treatment but demonstrated low/no efficacy in human clinical trials such as Doxorubicin [50], Curcumin [51], and 5-FU [52]. All drugs were added at their previously reported IC50 concentrations: 11nM Gemcitabine [53], 4.1nM Paclitaxel [54], 64 μM Cisplatin [47], 0.6 μM Doxorubicin [55], 13 μM 5-FU [56], and 17μM curcumin [57] to the cell particle complexes as well as the 2D cell cultures. The samples were further incubated for 2 days at 37°C following which the samples were washed well to remove residual drugs. The numbers of cell deaths in each group was tested using LDH assays (CytoTox96; PROMEGA, Madison, WI, USA), following manufacturers’ directions.

### Statistical analysis

The sample size used for all experiments was n = 4 unless mentioned otherwise. Statistical analysis was performed using one-way ANOVA with StatView software (V 5.0.1, SAS Institute Inc., Cary, NC). Values are presented as mean ± standard error. Statistical significance was considered when p<0.05.

## Results

### Microparticle characterization

The physical characteristics including particle size, surface charge, and polydispersity and pore diameter determined using ImageJ have been summarized in Table 1. The diameters of non-porous and porous (PLGA-Gelatin, PLGA-SBC, and PLGA-PMPs) microspheres were 26, 19, 34, and 42 μm respectively. All particles had zeta potential of -19mV or higher indicating that they are stable.

**Table 1:** Initial characterization of porous particles. Five images were analyzed per formulation.

Particle type	Diameter (μm)	Zeta potential (mV)	Pore Diameter (μm)
**Non-porous PLGA**	26±11	-19.3±0.3	-
**PLGA-gelatin**	19±5	-26.3±0.9	2.5±1.1 *
**PLGA-SBC**	34±13	-37.5±1.6	7.3±4.0 ^$^
**PLGA-PMPs**	42±10	-22.4±1.2	1.9±0.8*

*, ^$^ p<0.05 in comparison to pore diameter of PLGA-SBCs and PLGA-PMPs respectively

**Fig 1:**
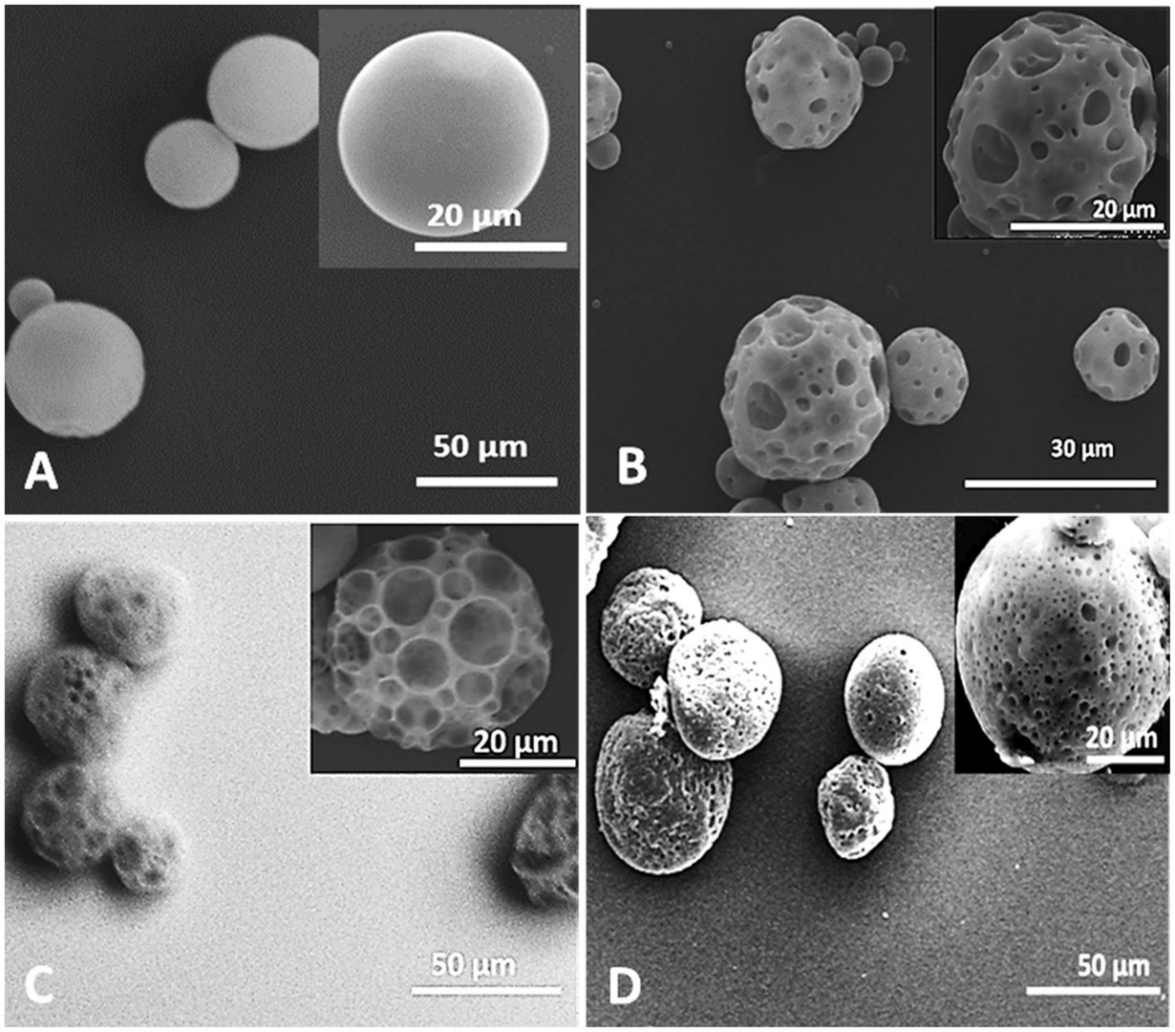
Morphology of porous PLGA MPs. SEM images of (A) non-porous PLGA MPs and porous MPs prepared using (B) gelatin, (C) SBC, and (D) PMPs as porogens. The spherical morphology and porous nature of PLGA-Gelatin, PLGA-SBC, and PLGA-PMPs can be clearly visualized.

SEM images indicated that non- porous PLGA particles (Fig 1A) had a smooth spherical surface, while PLGA-Gelatin (Fig 1B), PLGA-SBC (Fig 1C), and PLGA-PMPs (Fig 1D) microspheres were spherical with distinct pores present on the surface. ImageJ analysis established that PLGA-SBCs had an average pore diameter which was significantly larger in comparison to pore diameters of PLGA-SBC and PLGA-gelatin MPs.

### Stability and degradation kinetics

**Fig 2:**
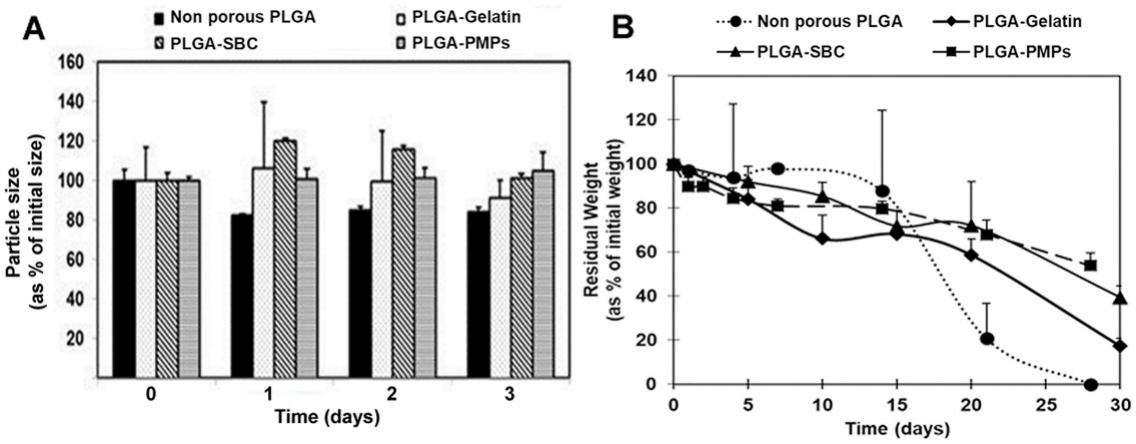
Physical characterization of porous PLGA MPs. (A) Particle stability in 10% serum indicating that non-porous PLGA, PLGA-SBC, PLGA-Gelatin, and PLGA-PMPs maintained their diameter and granulometric properties for 3 days. (B) Degradation of porous PLGA MPs investigated at 37°C for 4 weeks. PLGA-Gelatin particles degraded 83% of their initial weight in 28 days while PLGA-SBC particles and PLGA-PMPs were reduced to 61% and 46% of their original weight, respectively. The data plotted in terms of average ± standard error obtained from samples size (n) of 3.

The porous MPs were further characterized and compared with non-porous PLGA MPs in terms of their stability in media containing 10% serum. In media, all three types of particles were relatively stable and underwent minor fluctuations by about 20–30% of their original size, over a period of 3 days (Fig 2A). Furthermore, the degradation study conducted under physiological conditions for 4 weeks showed that the porous microspheres using gelatin, SBC, PMP porogens degraded 83%, 61%, and 46% of their original weight respectively, as shown in Fig 2B. On other hand, non-porous PLGA degraded completely within 4 weeks.

### Cell attachment studies

**Fig 3:**
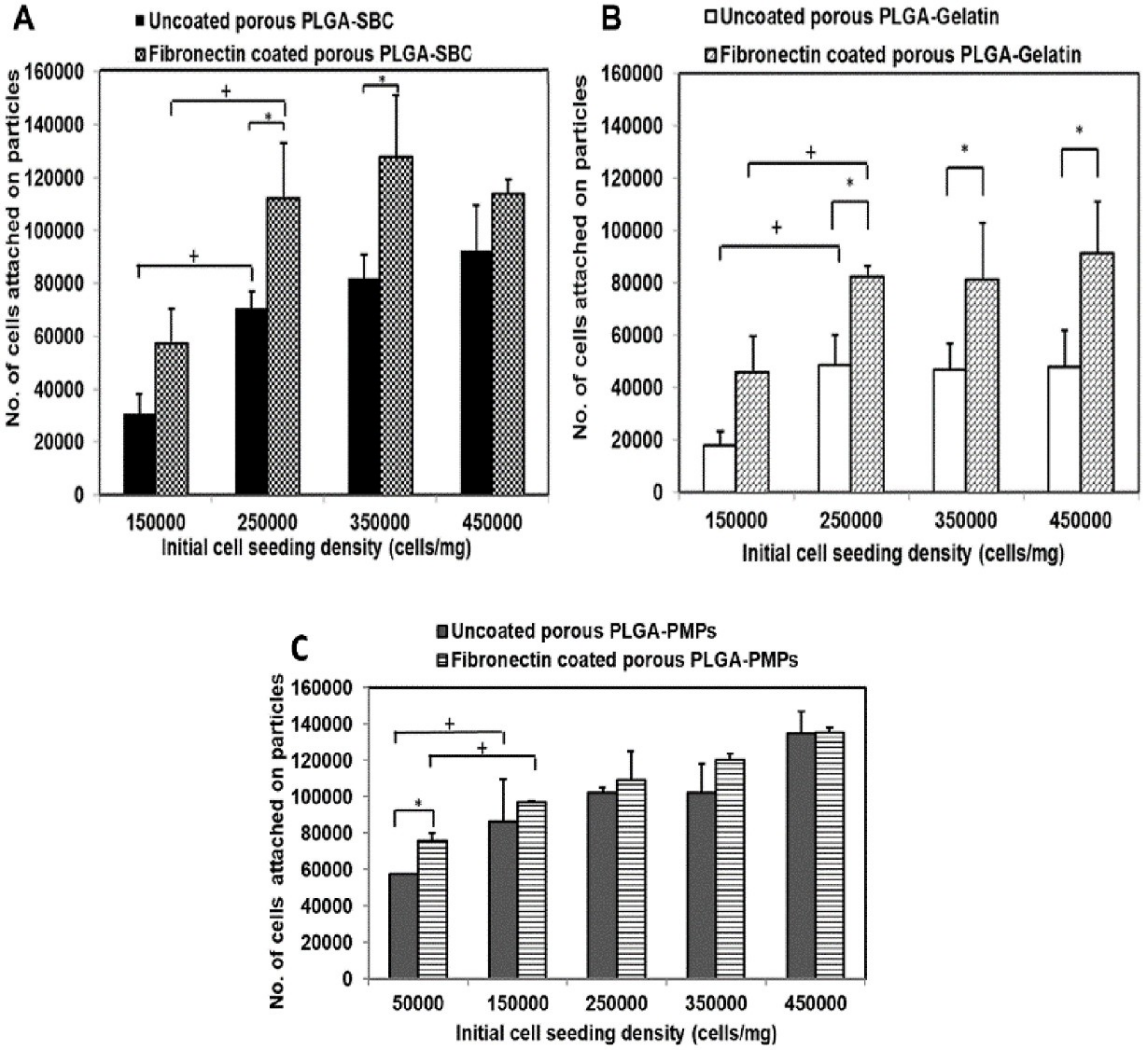
Cellular adhesion onto porous MPs. A549 cell attachment on uncoated and fibronectin-coated particles for 24 hours. Maximum cell attachment was observed at 250,000 cells/mg of particles cell density for (A) PLGA-SBC and (B) PLGA-gelatin particles. Cell attachment was saturated at 150,000 cells/mg density for (C) PLGA-PMPs. The data plotted in terms of average ± standard error obtained from samples size (n) of 4 (* represents p-value <0.05 w.r.t cell attachment on uncoated particles, + represents p- value <0.05 w.r.t cell attachment between different cell seeding densities).

*In vitro* studies were conducted to optimize the cell seeding density on the MPs and to compare cell binding affinity among all MPs. As shown in Fig 3A and 3B, cell attachment was saturated at a seeding density of 250,000 cells/ mg of PLGA-SBC and PLGA-Gelatin MPs with or without fibronectin coating (Fig 3A and 3B). The fibronectin coating resulted in enhanced cell attachment and proliferation compared to the uncoated particles. Interestingly, in the case of PLGA-PMPs, the saturation of cell attachment occurred at 150,000 cells/ mg seeding density (Fig 3C). No significant increase in cell attachment within each group was observed at densities >250,000 cells/ mg of MPs. When cell attachment on the different MPs at the same initial cell seeding density (250,000 cells/ mg MPs) was studied, PLGA-SBC MPs and PLGA-PMPs showed the highest average cell binding affinity (112,450 cells/mg and 109,372 cells/mg respectively) compared to PLGA-Gelatin MPs (82,725 cells/mg of particles).

**Fig 4:**
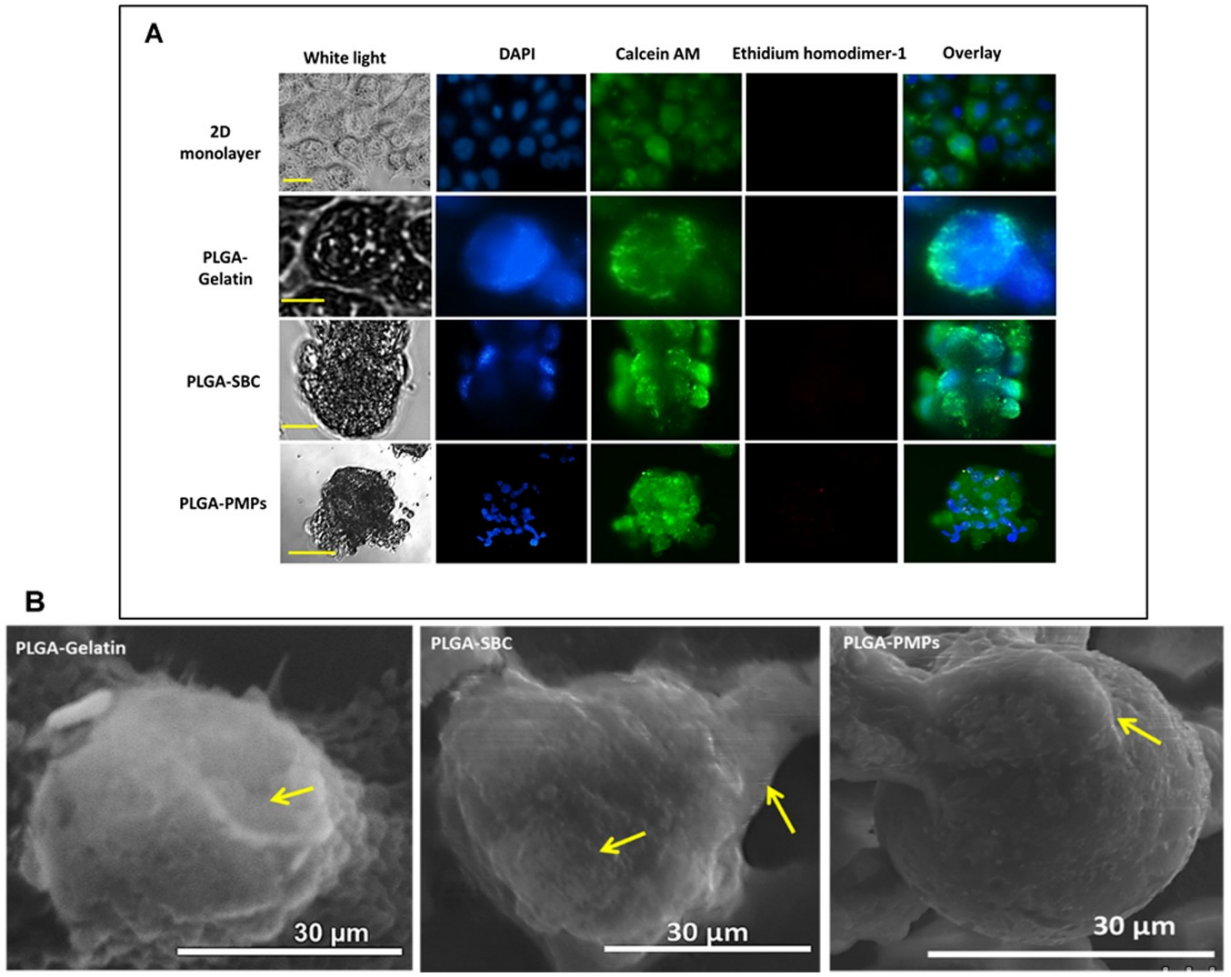
Cellular viability on porous MPs. (A) Live/dead and DAPI stained particles after 1-day culture indicating that the A549 lung cancer cells could attach onto the fibronectin-coated particles within 24 hours and were viable (green = live, red = dead, Scale = 10 μm). (B) SEM images of cell attachment on PLGA-Gelatin, PLGA-SBC and PLGA-PMPs demonstrate that the cells were clearly seen attached onto the surface of the MPs (arrows).

Further, cell attachment and viability (green) on our porous MPs was visualized using Live/Dead and DAPI staining. It was observed that the cells attached onto all particles within 24 hours with minimal cell death (red) (Fig 4A). Cell attachment on the fibronectin-coated MPs was visualized using SEM. The arrows indicate regions where the A549 cells have adhered onto the particles. The cells appear to have retained their morphology, and appendages can be seen indicating that the cells have spread out and begun to proliferate on the surface of all the MPs (Fig 4B). We do not find any difference in cell morphology and cell viability among all three MPs.

### Cell proliferation study

**Fig 5:**
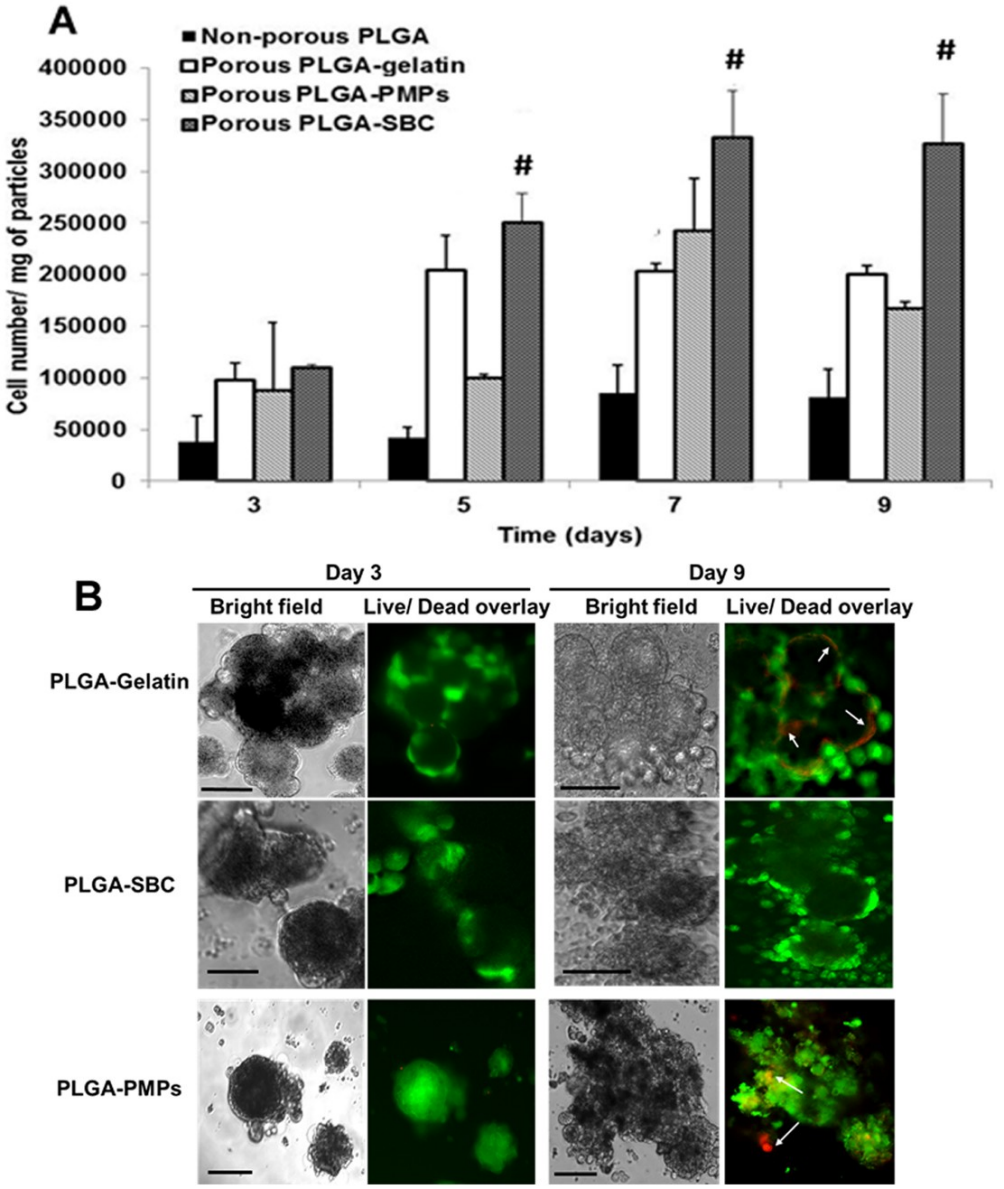
Cellular proliferation on porous MPs. (A) A549 cell proliferation on fibronectin-coated particles up to 9 days, showing significantly higher cell growth on PLGA-SBC porous particles compared to the non-porous control particles and other porous particles (PLGA-Gelatin, and PLGA-PMPs. The data plotted in terms of average ± standard error obtained from samples size (n) of 4 (# represents p<0.05 w.r.t porous PLGA-Gelatin and PLGA-PMPs). (B) Live/Dead staining shows A549 lung cancer cells attached on porous PLGA MPs were viable for up to 9 days with minimal cell death. PLGA-PMPs and PLGA-Gelatin MPs showed cell death on day 9 (arrows); however, cells on PLGA-SBC MPs remained viable throughout the study (Scale = 10 μm).

The cell proliferation study using A549 cells indicate that cell proliferation does occur on all three types of porous MPs, with greater cell growth observed on the porous MPs than on non-porous MPs (Fig 5A). However significantly higher cell proliferation occurred on the surface of porous PLGA-SBC MPs compared to proliferation on the other particles. Cell number appeared to saturate on PLGA-gelatin and PLGA-SBC MPs by days 5 and 7 respectively, indicating that the cells may not have any further surface available for proliferation. In addition, there is significant decrease in cell proliferation on PLGA-PMPs at day 9, as these particles have small pores, and hence oxygen and nutrient diffusion would be minimal. Our findings concur with the Live/Dead assay, where cell death could be distinctly seen on PLGA-PMPs and PLGA-gelatin by day 9; however, no significant cell death could be observed on PLGA-SBC particles up to day 9 (Fig 5B).

### *In vitro* drug screening

**Fig 6:**
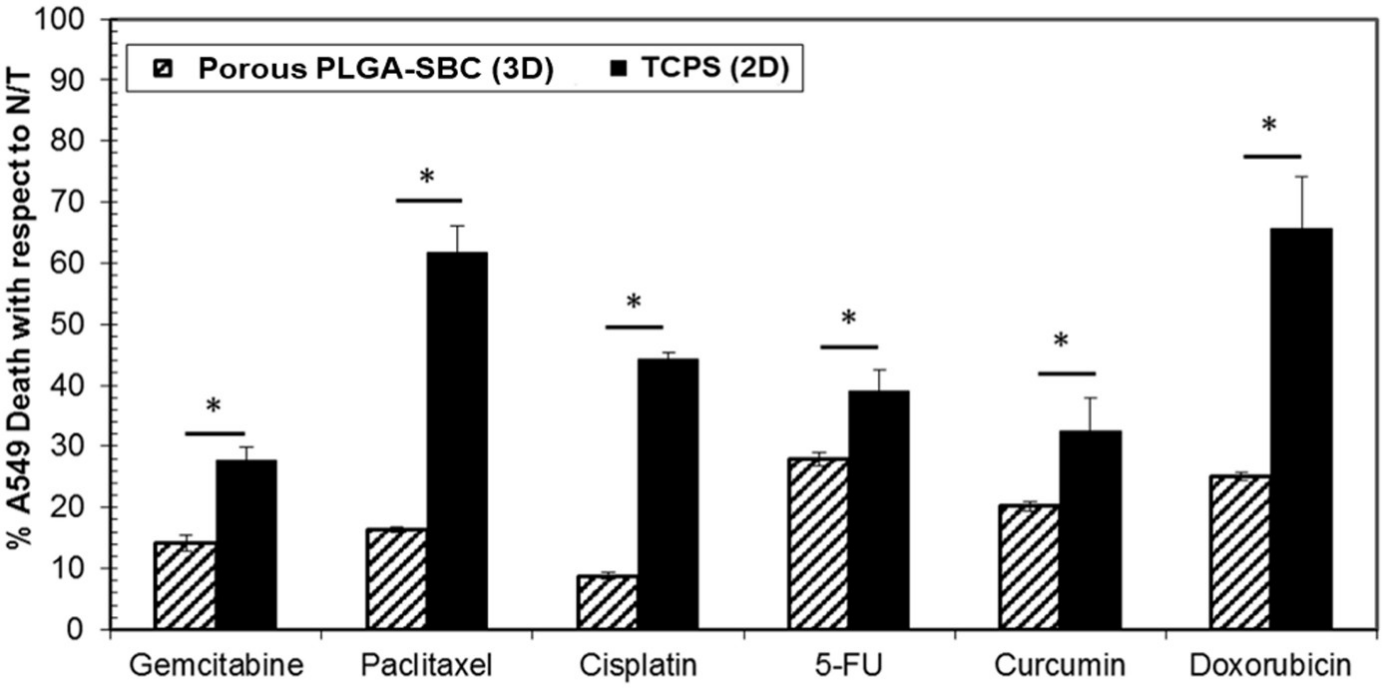
*In vitro* cancer drug screening. Effectiveness of chemotherapeutic drug treatment on A549 2D monolayer grown on TCPS and porous PLGA particles was quantified using LDH assays. It was observed that cancer drugs reduced only ~20–30% of A549 growth on the particles compared to its 2D counterparts when treated with the same concentrations of drugs. The percentage of A549 cell death was calculated with respect to untreated cells (N/T) lysed with triton. Data was statistically significant (n = 3, *p<0.05) when compared between two models (2D vs. particles) for each treatment groups.

Six different drugs, namely Gemcitabine, Paclitaxel, Cisplatin, 5-FU, Curcumin, and Doxorubicin, were screened *in vitro* for their therapeutic efficacies on 2D cell layers grown on tissue culture polystyrene (TCPS) and tumor aggregates formed using PLGA-SBC porous MPs. Both the cancer cell monolayer and the tumor aggregates were treated with IC_50_ concentrations of all drugs based on publications. As seen in Fig 6, at the same concentration all tested chemotherapeutic drugs caused greater cell death on the 2D cell monolayer compared to those cultured on the particles. These results confirm that A549 lung tumor cells cultured on different substrate conditions respond differently to chemotherapy drugs.

## Discussion

In this work, three types of porous PLGA MPs were prepared using different porogens, i.e. gelatin, SBC or the novel PMPs, to choose the optimal MP formulation with surface characteristics most suitable for lung cancer cell attachment and growth. Porous polymeric microparticle substrates have not been studied before for lung cancer cell culture *in vitro*. The most important finding of this work is that PLGA-SBC porous particles which had larger, more interconnected pores facilitated longer-term cell proliferation and viability than the other porous models indicating that pore diameter and porosity have direct implications on cell culture and viability on porous microparticle substrates. The lung cancer cells cultured on PLGA-SBC microparticles showed higher drug resistance compared to the 2D model at the same concentrations of cancer drugs, indicating that cells tend to respond differently to treatment depending on their arrangement. This is consistent with previously published reports comparing cell monolayers with cells cultured in a 3D format [5, 58]. Our results indicate the need for further investigation into PLGA-SBC porous particles-based tumor models and validating them *in vivo* to find the most promising model for pharmacological studies that will closely mimic reactions seen *in vivo*. We have also introduced a novel method of forming porous MPs using PMPs. The pores of these MPs can be tailored by varying the diameters of the PMPs used.

All porous MPs developed in this work were in the diameter range of 19–42 μm, which indicates that sufficient surface area will be available for A549 growth, since previously published works have shown that A549 cells have an average diameter of 6–15 μm [59, 60]. The standard deviations observed is typical of PLGA microspheres prepared using low amounts of PVA surfactant, as observed by other groups [61–63]. Ideally, a pore diameter of 20 μm or greater is desirable for growth and infiltration of mammalian cells, while smaller pores provide adequate surface area for cell attachment on the surface [64, 65]. The smaller pore diameter for PLGA-PMPs is due to the small diameter of PMP porogen, which is between 0.8 to 2.8 μm as described previously [35]. This novel porogen was tested to obtain pores with relatively uniform diameters distributed throughout the MP surface to allow uniform nutrient and oxygen diffusion. The SEM images confirm that PLGA-PMPs indeed have uniform pores of consistent diameters, compared to the other porous MPs.

The physical analysis of particles in this study also involved stability and degradation kinetics. The relatively unchanged sizes of all MPs suggest that they will be stable in serum and will not undergo major aggregation or clumping during initial cell seeding, which can reduce the surface area available for initial cell attachment and growth. It is also essential that the degradation rate of the scaffolds match the extracellular matrix deposition rate by the cells. The particles must maintain their integrity long enough for the cells to attach, proliferate and infiltrate into them [43], and should not undergo premature degradation. Our degradation studies show that at non-porous PLGA MPs degrade at much faster rate than its porous counterparts. Furthermore, PLGA-Gelatin, PLGA-SBC, PLGA-PMP MPs degraded 83%, 61% and 46% respectively of their original weight by 4 weeks. Several determinants including porosity and particle size account for the mechanism and rate of particle degradation [66]. Generally, PLGA degrade by hydrolysis of its ester linkages in presence of water. As these bonds are cleaved, the acidic degraded products observed to be accumulated within non-porous particles which further catalyze the polymer degradation itself. On other hand in MPs with open and interconnected pores, these oligomers diffused out and cleared from the vicinity of microscaffolds thereby diminishing its effect on the MPs. In support to our observation, Mittal et al, reported to observe inverse relationship of porosity on the degradation kinetics of PLGA MPs [67]. Similarly, Parmer et al. noted that porous microspheres resulted in 80–90% less degraded products relative to solid microspheres of similar diameter [68]. To conclude, due to the reduced presence of acidic monomers after MP degradation, the cellular response on porous microparticles could be superior to the smooth, non-porous ones.

Following physical characterization, the *in vitro* cell attachment and growth kinetics were optimized. The optimal density of 25 x 10^4^ cells per mg of particles observed by us concurs with results by Sahoo et al., where the very same cell seeding density was found to demonstrate improved MCF-7 breast cancer cell growth rate on their porous PLGA/polylactide (PLA) microspheres [43]. Fluorescent and SEM images have also confirmed that PLGA porous particles were able to support the A549 seeding and attachment. However, it was noted that PLGA-gelatin based microscaffolds could accommodate significantly lower number of cells than PLGA-SBC. This might be due to its small diameter that limit the surface area available for higher cell attachment. According to cell growth kinetics, porous microspheres promoted higher cell attachment and growth than smooth PLGA MPs, as expected and demonstrated by others [65, 69]. Almost two times more cells have attached and grown on porous microspheres than non-porous microspheres which clearly indicates the advantage of having porous structures on particles. These porous network can result in particles with surface which can enhance the cell adhesion and aid in cells to retain their natural morphology [70].

Among porous microspheres, the steady increase in cell proliferation up to 7 days observed by us agrees with previous studies [3, 21, 70, 71]. A549 growth on PLGA-SBC and PLGA–gelatin was similar until day 5; however, A549 continue to proliferate more on PLGA-SBC for up to 9 days. In case of PLGA-PMPs, A549 cells growth on these particles slowly increased over time for up to 7 days and a reduction in growth was observed after 9 days of culture. In agreement with our observation, Live/Dead assay also distinctly shows cell death on PLGA-gelatin and PLGA-PMPs particles on day 9; and better cell viability on PLGA-SBC. Mainly, two factors may have account the limited cell growth on PLGA-gelatin and PLGA-PMPs. First the particle diameter, the diameter of PLGA-gelatin was smaller than its other particle counterparts which results in lesser surface area available to the cells for attachment and proliferation. On other hand, PLGA-PMPs’ larger surface area allowed more space for initial cell attachment, spreading and growth. Second determinant is the pore diameter, large-diameter pores would help in efficient transport of nutrients and metabolites to support long term cell growth on microcarriers as noted by Lim et al [69]. Based on their study, polycaprolactone beads of pore diameters 25–50 μm and 50–100 μm demonstrated similar growth rate of chondrocytes up to 7 days, but after that time the beads with pore diameters of 50–100 μm showed significantly higher cell growth than those of 25–50μm. Although our porous particles have significantly smaller pore diameters than those PCL beads, their results on the effect of pore diameter on chondrocytes proliferation on microcarriers helps in understanding the reduced A549 growth on PLGA-gelatin and PLGA-PMPs (pore diameters <3 μm) by day 5 and day 9 of culture respectively than PLGA-SBCs (pore diameter >3 μm). In conclusion, the small size of particles as in case of PLGA-gelatin and small pore diameters in both PLGA-gelatin and PLGA-PMPs may have limited the nutrient/metabolite distribution; and thereby affected cell growth. Based on all the studies conducted, PLGA-SBC particles comparatively aids in greater cell attachment, proliferation and viability with time, than other particles.

A preliminary *in vitro* drug screening study was conducted on the lung tumor aggregates developed using PLGA-SBC particles, and compared to 2D models. Our results show that A549 cells grown on porous particles have higher resistance to chemotherapeutic drugs than cells grown on 2D culture. In concurrence with our findings, many other research works also reported increased drug resistance by the tumors when grown in 3D culture, than their 2D counterparts [72–75]. Unlike 2D models, cancer cells cultured on our porous PLGA-SBCs particles eventually aggregate to forms larger spheroid-like structures (as seen in Fig 5, after 3 and 9 days of culture) which may have relevance to tumors seen in *in vivo* and have resulted in similar observation with other 3D tumor models. Interactions of cancer cells with ECM protein (like fibronectin, collagen) were reported to prevent cell apoptosis and facilitate drug resistance [75, 76], which may have resulted in reduced cell death of A549 when cultured on fibronectin-coated PLGA-SBS porous particles. Other factors including limited drug penetration and poor uptake by the cells were commonly attributed to such resistance seen for tumor cells grown on 3D culture [73], may have also contributed to our observed results.

One interesting observation with the lung tumor aggregates developed using porous PLGA-SBC microparticles was that all drugs were less potent than with 2D cell cultures. Few previous published works, however, have shown differential drug responses between 2D and 3D models *in vitro* [5, 58]. For instance, Nirmalanandhan et al. tested the effect of chemotherapeutic drugs on *in vitro* 3D spheroids prepared using A549 and H358 lung cancer cells in collagen gel [58]. This group observed that Doxorubicin and Paclitaxel had lower therapeutic efficacy in 3D models than that of 2D monolayers. In addition, Gemcitabine efficiently inhibited the A549 growth on 3D tumor models more than on 2D cultures, and they found no difference in potency of Cisplatin in both cultures. The difference in cancer cell response to drug treatments may have occurred due to the different types of tumor models used in both experiments. Zanoni et al. investigated the potential source of variabilities that affects the reproducibility of data using tumor models [77]. One of the factors was heterogeneous distribution of the tumor as determined based on its volume and shape. The authors proposed an approach to pre-select the tumor spheroids such that they were homogeneous in nature, and this would prevent inconsistency in drug treatments using various *in vitro* tumor models.

However, our design of scaffold-based lung tumor aggregates has some limitations. Our porous particles were not porous enough to support cellular infiltration which helps in generating “three-dimensional” culture of lung cancer cells. We have utilized a simple mono-cell cultured tumor model to carry out proof-of-concept testing of the efficacy of anti-cancer compounds. Factors such as tumor architecture, interactions among cancer cells and with other stromal cells, and an acidic tumor microenvironment are major determinants of tumor growth *in vivo* [5]. In short, our lung tumor aggregates do not fully reflect the complex *in vivo* physiological conditions as well as incorporate all distinct tumor phenotypes. Therefore, our future studies will focus on incorporating these parameters in the development of tumor model, for more thorough comparison of drug responses with 2D monolayers. Also, we will perform detailed studies to validate proliferative and metabolic status of cells cultured on the porous polymeric matrix and compare it with *in vivo* results. In addition, we will further optimize the particles by increasing the pore diameter, co-culturing cancer cells with fibroblasts, adding more detailed drug screening studies and comparing with *in vivo* animal models.

Besides serving as a substrate for *in vitro* cell cultures, porous PLGA MPs are widely utilized to deliver therapeutics for disease treatment [78, 79] as well to transfer cells to injured tissue to promote regeneration [as reviewed in [37]]. For instance, Lee et al. delivered mesenchymal stem cells (MSCs) to a rat heart after myocardial infarction (MI) using polyethylenimine (PEI) modified porous PLGA microspheres; and demonstrated an improved *in vivo* engraftment rate of MSCs in infarcted myocardium than in cells alone, leading to enhanced artery blood flow, and they also reversed the adverse cardiac remodeling after MI [80]. Qutachi et al. on the other hand, treated porous PLGA microspheres with ethanolic sodium hydroxide solution to form scaffold structures in body temperature to use for bone tissue engineering [81]. Such sintered scaffolds offer multiscale porosity, micro- and macro porosity for cellular infiltration and growth with desired mechanical properties to meet the tissue needs. Also, 3D cell culture models could be used to assess the toxicity of nanomaterials as an alternative experiment for *in vivo* animal studies [82].

## Conclusions

In summary, biodegradable porous PLGA MPs prepared using three different porogens—gelatin, SBC and PMPs were synthesized and compared to determine the most promising formulation to be used as a substrate for *in vitro* lung tumor models. An innovative method of preparing porous MPs with uniform pores, using PMPs was also presented. Although all the three types of particles were stable and biodegradable, PLGA-SBC-based porous particles had relatively larger pores and better interconnectivity, and favored cell attachment, growth and viability more, and were thus chosen for further studies. Preliminary drug screening studies conducted using PLGA-SBC particles to determine the therapeutic efficacy of various lung cancer drugs demonstrated that the 3D tumor model responded differently to drugs of the same concentration, compared to the 2D cell monolayers. These results need to be confirmed with *in vivo* results to determine the most appropriate and comparatively more accurate model for future drug screening applications.

## References

[1] Mitra A, Mishra L, Li S. Technologies for deriving primary tumor cells for use in personalized cancer therapy. Trends in Biotechnology. 2013;31(6):347–54. 10.1016/j.tibtech.2013.03.006. 23597659 PMC3665643

[2] Romijn HJ. Development and advantages of serum-free, chemically defined nutrient media for culturing of nerve tissue. Biology of the Cell. 1988;63(3):263–8. 10.1111/j.1768-322X.1988.tb00749.x. 3066424

[3] Kang S-W, Bae YH. Cryopreservable and tumorigenic three-dimensional tumor culture in porous poly(lactic-co-glycolic acid) microsphere. Biomaterials. 2009;30(25):4227–32. 10.1016/j.biomaterials.2009.04.025. 19446875 PMC2760435

[4] Fischbach C, Chen R, Matsumoto T, Schmelzle T, Brugge JS, Polverini PJ, et al Engineering tumors with 3D scaffolds. Nat Methods. 2007;4(10):855–60. Epub 2007/09/04. 10.1038/nmeth1085. . 17767164

[5] Choi SY, Lin D, Gout PW, Collins CC, Xu Y, Wang Y. Lessons from patient-derived xenografts for better in vitro modeling of human cancer. Advanced drug delivery reviews. 2014;79–80:222–37. Epub 2014/10/12. 10.1016/j.addr.2014.09.009. . 25305336

[6] Hutchinson L, Kirk R. High drug attrition rates—where are we going wrong? Nat Rev Clin Oncol. 2011;8(4):189–90. Epub 2011/03/31. 10.1038/nrclinonc.2011.34. . 21448176

[7] Mehta G, Hsiao AY, Ingram M, Luker GD, Takayama S. Opportunities and challenges for use of tumor spheroids as models to test drug delivery and efficacy. Journal of controlled release: official journal of the Controlled Release Society. 2012;164(2):192–204. Epub 2012/05/23. 10.1016/j.jconrel.2012.04.045 . 22613880 PMC3436947

[8] Benien P, Swami A. 3D tumor models: history, advances and future perspectives. Future oncology (London, England). 2014;10(7):1311–27. Epub 2014/06/21. 10.2217/fon.13.274 . 24947267

[9] El Assal R, Gurkan UA, Chen P, Juillard F, Tocchio A, Chinnasamy T, et al 3-D microwell array system for culturing virus infected tumor cells. Scientific reports. 2016;6:39144 10.1038/srep39144 28004818 PMC5177905

[10] Asghar W, El Assal R, Shafiee H, Pitteri S, Paulmurugan R, Demirci U. Engineering cancer microenvironments for in vitro 3-D tumor models. Materials today (Kidlington, England). 2015;18(10):539–53. 10.1016/j.mattod.2015.05.002 . 28458612 PMC5407188

[11] Tocchio A, Durmus NG, Sridhar K, Mani V, Coskun B, El Assal R, et al Magnetically guided self‐assembly and coding of 3D living architectures. Advanced Materials. 2018;30(4):1705034. 10.1002/adma.201705034 PMC5847371 29215164

[12] Ricci C, Moroni L, Danti S. Cancer tissue engineering—new perspectives in understanding the biology of solid tumours—a critical review. OA Tissue Engineering. 2013;1(1):4 10.13172/2052-9643-1-1-607.

[13] Choi SW, Zhang Y, Xia Y. Three-dimensional scaffolds for tissue engineering: the importance of uniformity in pore size and structure. Langmuir. 2010;26(24):19001–6. Epub 2010/11/26. 10.1021/la104206h. . 21090781 PMC3005814

[14] Meng F, Meyer CM, Joung D, Vallera DA, McAlpine MC, Panoskaltsis‐Mortari A. 3D bioprinted in vitro metastatic models via reconstruction of tumor microenvironments. Advanced Materials. 2019;31(10):1806899. 10.1002/adma.201806899 PMC6996245 30663123

[15] Knight E, Przyborski S. Advances in 3D cell culture technologies enabling tissue‐like structures to be created in vitro. Journal of anatomy. 2015;227(6):746–56. 10.1111/joa.12257 25411113 PMC4694114

[16] Privalova AM, Uglanova SV, Kuznetsova NR, Klyachko NL, Golovin YI, Korenkov VV, et al Microencapsulated Multicellular Tumor Spheroids as a Tool to Test Novel Anticancer Nanosized Drug Delivery Systems In Vitro. Journal of Nanoscience and Nanotechnology. 2015;15(7):4806–14. 10.1166/jnn.2015.10508 26373041

[17] Yeung P, Sin HS, Chan S, Chan GCF, Chan BP. Microencapsulation of Neuroblastoma Cells and Mesenchymal Stromal Cells in Collagen Microspheres: A 3D Model for Cancer Cell Niche Study. PLOS ONE. 2015;10(12):e0144139 10.1371/journal.pone.0144139 26657086 PMC4682120

[18] Martinez-Ramos C, Lebourg M. Three-dimensional constructs using hyaluronan cell carrier as a tool for the study of cancer stem cells. J Biomed Mater Res B Appl Biomater. 2015;103(6):1249–57. Epub 2014/10/29. 10.1002/jbm.b.33304 . 25350680

[19] Yu L, Grist SM, Nasseri SS, Cheng E, Hwang YCE, Ni C, et al Core-shell hydrogel beads with extracellular matrix for tumor spheroid formation. Biomicrofluidics. 2015;9(2):024118 10.1063/1.4918754 25945144 PMC4401801

[20] Pradhan S, Clary JM, Seliktar D, Lipke EA. A three-dimensional spheroidal cancer model based on PEG-fibrinogen hydrogel microspheres. Biomaterials. 2017;115:141–54. 10.1016/j.biomaterials.2016.10.052. 27889665

[21] Horning JL, Sahoo SK, Vijayaraghavalu S, Dimitrijevic S, Vasir JK, Jain TK, et al 3-D Tumor Model for In Vitro Evaluation of Anticancer Drugs. Molecular Pharmaceutics. 2008;5(5):849–62. 10.1021/mp800047v 18680382

[22] Loh QL, Choong C. Three-dimensional scaffolds for tissue engineering applications: role of porosity and pore size. Tissue engineering Part B, Reviews. 2013;19(6):485–502. Epub 2013/06/25. 10.1089/ten.TEB.2012.0437 . 23672709 PMC3826579

[23] Wang M, Zhao Q. Electrospinning and Electrospray for Biomedical Applications. 2018.

[24] Fang J, Zhang Y, Yan S, Liu Z, He S, Cui L, et al Poly(l-glutamic acid)/chitosan polyelectrolyte complex porous microspheres as cell microcarriers for cartilage regeneration. Acta Biomaterialia. 2014;10(1):276–88. 10.1016/j.actbio.2013.09.002. 24025620

[25] Wei D-X, Dao J-W, Chen G-Q. A Micro-Ark for Cells: Highly Open Porous Polyhydroxyalkanoate Microspheres as Injectable Scaffolds for Tissue Regeneration. Advanced Materials. 2018;30(31):1802273 10.1002/adma.201802273 29920804

[26] Leong W, Wang D-A. Cell-laden Polymeric Microspheres for Biomedical Applications. Trends in Biotechnology. 2015;33(11):653–66. 10.1016/j.tibtech.2015.09.003 26475118

[27] Siegel RL, Miller KD, Jemal A. Cancer statistics, 2019. CA: A Cancer Journal for Clinicians. 2019;69(1):7–34. 10.3322/caac.21551 30620402

[28] Blandin Knight S, Crosbie PA, Balata H, Chudziak J, Hussell T, Dive C. Progress and prospects of early detection in lung cancer. Open biology. 2017;7(9):170070 10.1098/rsob.170070 . 28878044 PMC5627048

[29] Movia D, Bazou D, Volkov Y, Prina-Mello A. Multilayered Cultures of NSCLC cells grown at the Air-Liquid Interface allow the efficacy testing of inhaled anti-cancer drugs. Scientific Reports. 2018;8(1):12920 10.1038/s41598-018-31332-6 30150787 PMC6110800

[30] Justin AW, Brooks RA, Markaki AE. Multi-casting approach for vascular networks in cellularized hydrogels. Journal of the Royal Society, Interface. 2016;13(125):20160768 10.1098/rsif.2016.0768 . 27928031 PMC5221527

[31] Bin X, Darren R, Shakir L, Xiaoshu Z, John PS, Laurie A, et al Patterning cellular compartments within TRACER cultures using sacrificial gelatin printing. Biofabrication. 2016;8(3):035018 10.1088/1758-5090/8/3/035018 27631341

[32] Choi S-W, Yeh Y-C, Zhang Y, Sung H-W, Xia Y. Uniform beads with controllable pore sizes for biomedical applications. Small (Weinheim an der Bergstrasse, Germany). 2010;6(14):1492–8. 10.1002/smll.201000544 . 20578116 PMC3011368

[33] Annabi N, Nichol JW, Zhong X, Ji C, Koshy S, Khademhosseini A, et al Controlling the porosity and microarchitecture of hydrogels for tissue engineering. Tissue engineering Part B, Reviews. 2010;16(4):371–83. Epub 2010/03/17. 10.1089/ten.TEB.2009.0639 . 20121414 PMC2946907

[34] Choi S-W, Zhang Y, Yeh Y-C, Wooten AL, Xia Y. Biodegradable porous beads and their potential applications in regenerative medicine. Journal of Materials Chemistry. 2012;22(23):11442–51.

[35] Nguyen KT, Shukla KP, Moctezuma M, Braden ARC, Zhou J, Hu Z, et al Studies of the cellular uptake of hydrogel nanospheres and microspheres by phagocytes, vascular endothelial cells, and smooth muscle cells. Journal of biomedical materials research Part A. 2009;88(4):1022–30. 10.1002/jbm.a.31734 . 18404709 PMC2908386

[36] Koppolu B, Rahimi M, Nattama S, Wadajkar A, Nguyen KT. Development of multiple-layer polymeric particles for targeted and controlled drug delivery. Nanomedicine: nanotechnology, biology, and medicine. 2010;6(2):355–61. Epub 2009/08/25. 10.1016/j.nano.2009.07.008. . 19699325 PMC2881641

[37] Huang CC, Wei HJ, Yeh YC, Wang JJ, Lin WW, Lee TY, et al Injectable PLGA porous beads cellularized by hAFSCs for cellular cardiomyoplasty. Biomaterials. 2012;33(16):4069–77. Epub 2012/03/06. 10.1016/j.biomaterials.2012.02.024. . 22386922

[38] Ke CJ, Chiang WL, Liao ZX, Chen HL, Lai PS, Sun JS, et al Real-time visualization of pH-responsive PLGA hollow particles containing a gas-generating agent targeted for acidic organelles for overcoming multi-drug resistance. Biomaterials. 2013;34(1):1–10. Epub 2012/10/10. 10.1016/j.biomaterials.2012.09.023. . 23044041

[39] Menon JU, Ravikumar P, Pise A, Gyawali D, Hsia CC, Nguyen KT. Polymeric nanoparticles for pulmonary protein and DNA delivery. Acta Biomater. 2014;10(6):2643–52. Epub 2014/02/12. 10.1016/j.actbio.2014.01.033 . 24512977 PMC4008694

[40] Ravikumar P, Menon JU, Punnakitikashem P, Gyawali D, Togao O, Takahashi M, et al Nanoparticle facilitated inhalational delivery of erythropoietin receptor cDNA protects against hyperoxic lung injury. Nanomedicine. 2016;12(3):811–21. Epub 2015/11/01. 10.1016/j.nano.2015.10.004 . 26518603 PMC4809756

[41] Wadajkar AS, Menon JU, Tsai Y-S, Gore C, Dobin T, Gandee L, et al Prostate cancer-specific thermo-responsive polymer-coated iron oxide nanoparticles. Biomaterials. 2013;34(14):3618–25. 10.1016/j.biomaterials.2013.01.062 . 23419645

[42] Kona S, Dong J-F, Liu Y, Tan J, Nguyen KT. Biodegradable nanoparticles mimicking platelet binding as a targeted and controlled drug delivery system. International journal of pharmaceutics. 2012;423(2):516–24. Epub 2011/12/06. 10.1016/j.ijpharm.2011.11.043 . 22172292 PMC3273581

[43] Sahoo SK, Panda AK, Labhasetwar V. Characterization of porous PLGA/PLA microparticles as a scaffold for three dimensional growth of breast cancer cells. Biomacromolecules. 2005;6(2):1132–9. 10.1021/bm0492632. 15762686

[44] Sylvester PW. Optimization of the tetrazolium dye (MTT) colorimetric assay for cellular growth and viability. Drug design and discovery: Springer; 2011 p. 157–68. 10.1007/978-1-61779-012-6_9 21318905

[45] Cole S. Rapid chemosensitivity testing of human lung tumor cells using the MTT assay. Cancer chemotherapy and pharmacology. 1986;17(3):259–63. 3742711 10.1007/BF00256695

[46] Voskoglou-Nomikos T, Pater JL, Seymour L. Clinical predictive value of the in vitro cell line, human xenograft, and mouse allograft preclinical cancer models. Clinical cancer research: an official journal of the American Association for Cancer Research. 2003;9(11):4227–39. Epub 2003/10/02. Not Found. . 14519650

[47] Zhang P, Gao WY, Turner S, Ducatman BS. Gleevec (STI-571) inhibits lung cancer cell growth (A549) and potentiates the cisplatin effect in vitro. Molecular cancer. 2003;2(1):1 10.1186/1476-4598-2-1. 12537587 PMC149413

[48] Scarpace SL. Metastatic squamous cell non-small-cell lung cancer (NSCLC): disrupting the drug treatment paradigm with immunotherapies. Drugs in context. 2015;4:212289 Epub 2015/11/18. 10.7573/dic.212289. . 26576187 PMC4630973

[49] Yang JC, Wu YL, Schuler M, Sebastian M, Popat S, Yamamoto N, et al Afatinib versus cisplatin-based chemotherapy for EGFR mutation-positive lung adenocarcinoma (LUX-Lung 3 and LUX-Lung 6): analysis of overall survival data from two randomised, phase 3 trials. The Lancet Oncology. 2015;16(2):141–51. Epub 2015/01/16. 10.1016/s1470-2045(14)71173-8. . 25589191

[50] Otterson GA, Villalona-Calero MA, Hicks W, Pan X, Ellerton JA, Gettinger SN, et al Phase I/II study of inhaled doxorubicin combined with platinum-based therapy for advanced non-small cell lung cancer. Clinical cancer research: an official journal of the American Association for Cancer Research. 2010;16(8):2466–73. Epub 2010/04/08. 10.1158/1078-0432.ccr-09-3015. . 20371682 PMC4262532

[51] Alexandrow MG, Song LJ, Altiok S, Gray J, Haura EB, Kumar NB. Curcumin: a novel Stat3 pathway inhibitor for chemoprevention of lung cancer. European journal of cancer prevention: the official journal of the European Cancer Prevention Organisation (ECP). 2012;21(5):407–12. Epub 2011/12/14. 10.1097/CEJ.0b013e32834ef194. . 22156994 PMC3319490

[52] Zhao JG, Ren KM, Tang J. Overcoming 5-Fu resistance in human non-small cell lung cancer cells by the combination of 5-Fu and cisplatin through the inhibition of glucose metabolism. Tumour biology: the journal of the International Society for Oncodevelopmental Biology and Medicine. 2014;35(12):12305–15. Epub 2014/09/28. 10.1007/s13277-014-2543-3. . 25260882

[53] Edelman M, Quam H, Mullins B. Interactions of gemcitabine, carboplatin and paclitaxel in molecularly defined non-small-cell lung cancer cell lines. Cancer Chemotherapy and Pharmacology. 2001;48(2):141–4. 10.1007/s002800000273. 11561780

[54] Liebmann J, Cook J, Lipschultz C, Teague D, Fisher J, Mitchell J. Cytotoxic studies of paclitaxel (Taxol) in human tumour cell lines. British journal of cancer. 1993;68(6):1104 10.4172/2155-983X.1000e144. 7903152 PMC1968657

[55] Xin Y, Yin F, Qi S, Shen L, Xu Y, Luo L, et al Parthenolide reverses doxorubicin resistance in human lung carcinoma A549 cells by attenuating NF-kB activation and HSP70 up-regulation. Toxicology Letters. 2013;221(2):73–82. 10.1016/j.toxlet.2013.06.215. 23792430

[56] Libby P, DiCarli M, Weissleder R. The Vascular Biology of Atherosclerosis and Imaging Targets. Journal of Nuclear Medicine. 2010;51(Supplement 1):33S–7S. 10.2967/jnumed.109.069633 20395349

[57] Amin AR, Haque A, Rahman MA, Chen ZG, Khuri FR, Shin DM. Curcumin induces apoptosis of upper aerodigestive tract cancer cells by targeting multiple pathways. PLoS One. 2015;10(4):e0124218 Epub 2015/04/25. 10.1371/journal.pone.0124218 . 25910231 PMC4409383

[58] Nirmalanandhan VS, Duren A, Hendricks P, Vielhauer G, Sittampalam GS. Activity of anticancer agents in a three-dimensional cell culture model. Assay and drug development technologies. 2010;8(5):581–90. 10.1089/adt.2010.0276. 20662735

[59] Tanaka M, Inase N, Fushimi K, Ishibashi K, Ichioka M, Sasaki S, et al Induction of aquaporin 3 by corticosteroid in a human airway epithelial cell line. American Journal of Physiology—Lung Cellular and Molecular Physiology. 1997;273(5):L1090–L5. Not found. 10.1152/ajplung.1997.273.5.L1090 9374739

[60] Jiang RD, Shen H, Piao YJ. The morphometrical analysis on the ultrastructure of A549 cells. Rom J Morphol Embryol. 2010;51(4):663–7. Epub 2010/11/26. https://doi.org/510410663667. . 21103623

[61] Choi Y-S, Joo J-R, Hong A, Park J-S. Development of drug-loaded PLGA microparticles with different release patterns for prolonged drug delivery. Bulletin of the Korean Chemical Society. 2011;32(3):867–72.

[62] Kirby GT, White LJ, Rahman CV, Cox HC, Qutachi O, Rose FR, et al PLGA-based microparticles for the sustained release of BMP-2. Polymers. 2011;3(1):571–86.

[63] Kemala T, Budianto E, Soegiyono B. Preparation and characterization of microspheres based on blend of poly (lactic acid) and poly (ɛ-caprolactone) with poly (vinyl alcohol) as emulsifier. Arabian Journal of Chemistry. 2012;5(1):103–8.

[64] Chou M-J, Hsieh C-H, Yeh P-L, Chen P-C, Wang C-H, Huang Y-Y. Application of open porous poly(D,L-lactide-co-glycolide) microspheres and the strategy of hydrophobic seeding in hepatic tissue cultivation. Journal of Biomedical Materials Research Part A. 2013;101(10):2862–9. 10.1002/jbm.a.34594. 23505008

[65] Zhang T, Zhang Q, Chen J, Fang K, Dou J, Gu N. The controllable preparation of porous PLGA microspheres by the oil/water emulsion method and its application in 3D culture of ovarian cancer cells. 2014;452:115–24. 10.1016/j.colsurfa.2014.03.085.

[66] Klose D, Siepmann F, Elkharraz K, Krenzlin S, Siepmann J. How porosity and size affect the drug release mechanisms from PLGA-based microparticles. International Journal of Pharmaceutics. 2006;314(2):198–206. 10.1016/j.ijpharm.2005.07.031. 16504431

[67] Mittal A, Negi P, Garkhal K, Verma S, Kumar N. Integration of porosity and bio-functionalization to form a 3D scaffold: cell culture studies and in vitro degradation. Biomedical materials (Bristol, England). 2010;5(4):045001 Epub 2010/06/12. 10.1088/1748-6041/5/4/045001. . 20539055

[68] Parmar N, Ahmadi R, Day RM. A Novel Method for Differentiation of Human Mesenchymal Stem Cells into Smooth Muscle-Like Cells on Clinically Deliverable Thermally Induced Phase Separation Microspheres. Tissue Engineering Part C, Methods. 2015;21(4):404–12. 10.1089/ten.tec.2014.0431. 25205072 PMC4382826

[69] Lim SM, Lee HJ, Oh SH, Kim JM, Lee JH. Novel fabrication of PCL porous beads for use as an injectable cell carrier system. Journal of biomedical materials research Part B, Applied biomaterials. 2009;90(2):521–30. Epub 2009/01/16. 10.1002/jbm.b.31313. . 19145632

[70] Zhang Q, Tan K, Ye Z, Zhang Y, Tan W, Lang M. Preparation of open porous polycaprolactone microspheres and their applications as effective cell carriers in hydrogel system. Materials Science and Engineering: C. 2012;32(8):2589–95. 10.1016/j.msec.2012.07.045.

[71] Kang SW, Seo SW, Choi CY, Kim BS. Porous poly(lactic-co-glycolic acid) microsphere as cell culture substrate and cell transplantation vehicle for adipose tissue engineering. Tissue Eng Part C Methods. 2008;14(1):25–34. Epub 2008/05/06. 10.1089/tec.2007.0290. . 18454643

[72] Doillon CJ, Gagnon E, Paradis R, Koutsilieris M. Three-dimensional culture system as a model for studying cancer cell invasion capacity and anticancer drug sensitivity. Anticancer research. 2004;24(4):2169–77. Epub 2004/08/28. Not found. . 15330157

[73] Godugu C, Patel AR, Desai U, Andey T, Sams A, Singh M. AlgiMatrix Based 3D Cell Culture System as an In-Vitro Tumor Model for Anticancer Studies. PLOS ONE. 2013;8(1):e53708 10.1371/journal.pone.0053708. 23349734 PMC3548811

[74] Ruppen J, Cortes-Dericks L, Marconi E, Karoubi G, Schmid RA, Peng R, et al A microfluidic platform for chemoresistive testing of multicellular pleural cancer spheroids. Lab on a Chip. 2014;14(6):1198–205. 10.1039/C3LC51093J. 24496222

[75] Wang D-D, Liu W, Chang J-J, Cheng X, Zhang X-Z, Xu H, et al Bioengineering three-dimensional culture model of human lung cancer cells: an improved tool for screening EGFR targeted inhibitors. RSC Advances. 2016;6(29):24083–90. 10.1039/C6RA00229C.

[76] Sethi T, Rintoul RC, Moore SM, MacKinnon AC, Salter D, Choo C, et al Extracellular matrix proteins protect small cell lung cancer cells against apoptosis: a mechanism for small cell lung cancer growth and drug resistance in vivo. Nature medicine. 1999;5(6):662–8. Epub 1999/06/17. 10.1038/9511. . 10371505

[77] Zanoni M, Piccinini F, Arienti C, Zamagni A, Santi S, Polico R, et al 3D tumor spheroid models for in vitro therapeutic screening: a systematic approach to enhance the biological relevance of data obtained. Scientific Reports. 2016;6:19103 10.1038/srep19103. 26752500 PMC4707510

[78] Cai Y, Chen Y, Hong X, Liu Z, Yuan W. Porous microsphere and its applications. International Journal of Nanomedicine. 2013;8:1111–20. 10.2147/IJN.S41271. 23515359 PMC3600995

[79] Wang C, Wu D, Yang J, Han H, Xing Z, Zhang Y, et al Porous PLGA microparticles to encapsulate doxorubicin and polyethylenimine/miR-34a for inhibiting the proliferation and migration of lung cancer. RSC Advances. 2015;5(99):81445–8. 10.1039/C5RA15516A.

[80] Lee YS, Joo WS, Kim HS, Kim SW. Human Mesenchymal Stem Cell Delivery System Modulates Ischemic Cardiac Remodeling With an Increase of Coronary Artery Blood Flow. Mol Ther. 2016;24(4):805–11. 10.1038/mt.2016.22. 26782638 PMC4886938

[81] Qutachi O, Vetsch JR, Gill D, Cox H, Scurr DJ, Hofmann S, et al Injectable and porous PLGA microspheres that form highly porous scaffolds at body temperature. Acta Biomaterialia. 2014;10(12):5090–8. 10.1016/j.actbio.2014.08.015. 25152354 PMC4226323

[82] Dubiak-Szepietowska M, Karczmarczyk A, Jonsson-Niedziolka M, Winckler T, Feller KH. Development of complex-shaped liver multicellular spheroids as a human-based model for nanoparticle toxicity assessment in vitro. Toxicology and applied pharmacology. 2016;294:78–85. Epub 2016/01/31. 10.1016/j.taap.2016.01.016. . 26825373

